# High pressure thermal inactivation of *Clostridium botulinum* type E endospores – kinetic modeling and mechanistic insights

**DOI:** 10.3389/fmicb.2015.00652

**Published:** 2015-07-03

**Authors:** Christian A. Lenz, Kai Reineke, Dietrich Knorr, Rudi F. Vogel

**Affiliations:** ^1^Lehrstuhl für Technische Mikrobiologie, Technische Universität MünchenFreising, Germany; ^2^Quality and Safety of Food and Feed, Leibniz Institute for Agricultural Engineering (ATB), PotsdamGermany; ^3^Department of Food Biotechnology and Food Process Engineering, Technische Universität BerlinBerlin, Germany

**Keywords:** high pressure thermal (HPT) processing, pressure-assisted thermal sterilization (PATS), bacterial endospores, *Clostridium botulinum* type E, food safety, inactivation kinetics, kinetic modeling, dipicolinic acid release

## Abstract

Cold-tolerant, neurotoxigenic, endospore forming *Clostridium (C.) botulinum* type E belongs to the non-proteolytic physiological *C. botulinum* group II, is primarily associated with aquatic environments, and presents a safety risk for seafood. High pressure thermal (HPT) processing exploiting the synergistic effect of pressure and temperature can be used to inactivate bacterial endospores. We investigated the inactivation of *C. botulinum* type E spores by (near) isothermal HPT treatments at 300–1200 MPa at 30–75°C for 1 s to 10 min. The occurrence of heat and lysozyme susceptible spore fractions after such treatments was determined. The experimental data were modeled to obtain kinetic parameters and represented graphically by isoeffect lines. In contrast to findings for spores of other species and within the range of treatment parameters applied, zones of spore stabilization (lower inactivation than heat treatments alone), large heat susceptible (HPT-induced germinated) or lysozyme-dependently germinable (damaged coat layer) spore fractions were not detected. Inactivation followed first order kinetics. Dipicolinic acid release kinetics allowed for insights into possible inactivation mechanisms suggesting a (poorly effective) physiologic-like (similar to nutrient-induced) germination at ≤450 MPa/≤45°C and non-physiological germination at >500 MPa/>60–70°C. Results of this study support the existence of some commonalities in the HPT inactivation mechanism of *C. botulinum* type E spores and *Bacillus* spores although both organisms have significantly different HPT resistance properties. The information presented here contributes to closing the gap in knowledge regarding the HPT inactivation of spore formers relevant to food safety and may help industrial implementation of HPT processing. The markedly lower HPT resistance of *C. botulinum* type E spores compared with the resistance of spores from other *C. botulinum* types could allow for the implementation of milder processes without endangering food safety.

## Introduction

*Clostridium botulinum* type E belongs to the non-proteolytic *C. botulinum* group II, one of four phylogenetically distinct lineages comprising several different species (Collins and East, 1998). Endospores of this organism are primarily associated with aquatic environments including sea and fresh water sediments in temperate regions of the northern hemisphere (Hielm et al., 1998a,b). Consequently, seafood can be contaminated with *C. botulinum* type E (Hyytia et al., 1998; Hyytiä-Trees, 1999). Although its spores are more susceptible to inactivation by heat than those from other *C. botulinum* types, they can survive mild heat treatments such as traditional hot-smoking processes, where the internal product temperature typically peaks at 65–85°C (Hyytia et al., 1998; Hyytiä-Trees, 1999). Outgrowth may lead to the formation of the highly potent Botulinum neurotoxin, BoNT/E (Peck et al., 2008), causing flaccid paralysis (botulism), which may lead to death (Lund and Peck, 2000; Peck, 2009). The fact that *C. botulinum* type E is able to grow and form toxin at temperatures as low as 3°C (Peck, 2006) increases the hazards arising from this organism, especially for sous-vide products and other refrigerated processed foods of extended durability (REPFEDs) where food safety relies on mild processing conditions and refrigerated storage (Lund and Peck, 1994).

Whereas pressure-assisted thermal sterilization (PATS) is currently used to rapidly and homogenously reach the sterilization temperature of 121.1°C in a food product exploiting adiabatic heating effects, high pressure thermal (HPT) processing at temperatures below the sterilization temperature is not yet used in an industrial scale (Reineke et al., 2013a). One important reason for this can be found in the design of suitable and reliable HPT processes (robust equipment and precise process control). Especially, large variations in temperature in the pressure vessel, which can occur depending on the process parameters (extended dwell times), vessel design (large vessels), and thermodynamic properties of the vessel wall and any material in the vessel, present a major challenge in the industrial implementation of HPT (and PATS) processes. However, being able to lower the process target temperature without endangering food safety would make it possible to reduce unwanted effects on nutritionally valuable molecules (e.g., vitamins), appearance (color, texture), and taste of processed food (Heinz and Buckow, 2010). Additionally, the use of lower process temperatures might be advantageous in terms of the required robustness of high pressure equipment, material fatigue, and for loading and unloading during a typical operation.

Another major factor impeding the scale-up of HPT processing can be found in the gap in knowledge regarding the behavior of bacterial endospores treated by high pressure. The fact that (i) *C. botulinum* type E presents the safety determinant for fishery products, (ii) such products are frequently very sensitive to heat, and (iii) *C. botulinum* type E spores are less resistant to HPT treatments than those from many other spore formers including other *C. botulinum* serotypes (Reddy et al., 1999; Reddy et al., 2003, 2006, 2010; Lenz and Vogel, 2014, 2015; Skinner et al., 2014), makes HPT processing a promising technology for the production of safe and stable food with acceptable sensorial and nutritional characteristics (Campus, 2010; Erkan et al., 2011; Sevenich et al., 2015).

However, despite of some useful insights in the HPT-mediated inactivation mechanism of spores of the well-characterized model organism *Bacillus subtilis* (Reineke et al., 2013a), knowledge on spores from other species including important food intoxicating organisms is scarce. The aim of this study was to contribute to closing this gap by investigating the inactivation of *C. botulinum* type E in a broad range of pressures (300–1200 MPa) combined with temperatures of 30–75°C applied for dwell times of 1–600 s. Short (1 s) treatments were included and HPT processes were designed to ensure treatments under (near) isothermal-isobaric conditions to facilitate the comparability of results from different high pressure units (Reineke et al., 2012). Additionally, (a) dipicolinic acid (DPA) release, (b) heat susceptible, and (c) lysozyme-dependently germinable spore fractions (i.e., spores, which only germinate when lysozyme is present in the recovery medium) after HPT treatments were determined, which had several reasons:

(a) The release of DPA was reported to present the rate-limiting step in the inactivation of *B. subtilis* spores and to occur in a pressure-, temperature-, and time-dependent manner (Reineke et al., 2013a), and DPA release kinetics are presumed to allow for drawing conclusions on mechanisms underlying spore inactivation by HPT treatments.
(b) The heat treatment of pressurized spore samples was previously employed to estimate the number of spores, which survived HPT treatments but lost their spore-specific heat resistance (e.g., Reineke et al., 2013b). This is likely to be the case for spores, which underwent high pressure-induced physiologic-like (similar to nutrient-induced) germination, which can be triggered by relatively low pressure levels in combination with mild processing temperatures (typically at around 150 MPa at ambient temperatures for *B. subtilis*, Gould and Sale, 1970; Reineke et al., 2012). Thus, enumerating the number of spores surviving a second thermal treatment completes DPA release data providing further information on possible HPT inactivation mechanisms. Additionally, the efficiency of two-step inactivation processes employing a mild heating step after HPT processing of food can be estimated.
(c) Lysozyme in the recovery medium was reported to have a significant impact on the outcome of studies investigating the heat and HPT inactivation of *C. botulinum* spores (Alderton et al., 1974; Peck et al., 1992; Lindstrom et al., 2003). Additionally, *c*- and/or *g*-type lysozyme can be commonly found in various fish species (Saurabh and Sahoo, 2008; Callewaert and Michiels, 2010) including many edible fish (Dautigny et al., 1991; Fujiki et al., 2000; Kyomuhendo et al., 2007; Larsen et al., 2009) and may remain active after mild heat treatments (Proctor and Cunningham, 1988). Thus, viable spore counts determined in the absence of lysozyme could lead to an underestimation of the risk arising from *C. botulinum*. Additionally, lysozyme in the recovery medium enables the detection of spores with both defects in their cortex lytic machinery and coat layers but without severe damages in other spore components or molecules essential for germination and outgrowth. This provides additional information on the nature of damages HPT treatments provoke.

Kinetic data obtained in this study was used to model isoeffect lines for spore inactivation, heat susceptible, and lysozyme-dependently germinable fractions of spore populations as well as for HPT-mediated DPA release. This facilitates the description of spore destruction, the visualization of trends and interconnections, and drawing conclusions on mechanisms underlying spore inactivation.

## Materials and Methods

### Strains and Spore Suspension Preparation

Three *C. botulinum* type E strains, TMW 2.990 (Beluga, NCBI accession: NZ_ACSC00000000), TMW 2.992 (1576, Johannsen, 1963), and TMW 2.994 (Baumgart, 1972) were used. Strains, growth conditions, harvesting, and spore suspension purification were essentially done as previously described (Lenz and Vogel, 2015). Briefly, strains were grown from glycerol stocks in TPYC (tryptone, peptone, yeast extract, sugar mix (glucose, maltose, cellobiose, soluble starch)) medium (Artin et al., 2008) for 24 ± 2 h at 28 °C. Growing cultures were used to inoculate SFE (sediment fish extract) sporulation medium, which partially resembles the natural environment of *C. botulinum* type E and appears suitable for the production of highly resistant spores (Lenz and Vogel, 2014). Spores were grown under anaerobic conditions (85% N_2_, 10% CO_2_, 5% H_2_) at 20°C, a sporulation temperature contributing to the production of resistant spores (Lenz and Vogel, 2015), for 12 ± 1 days and harvested by centrifugation. Three wash cycles {one in distilled water, one in S+ [0.85% saline + 0.1% Antifoam B Emulsion (Dow Corning, Germany)], and one in distilled water} were followed by an ethanol treatment (2 h at room temperature) and another three wash cycles in distilled water. Spores were resuspended in IPB (imidazole phosphate buffer, pH 7) to given an initial viable spore count of ∼10^7^/mL. Spore suspensions containing >99% phase bright spores were stored at 4°C until treated (maximum 7 days).

### High Pressure Equipment and Sample Packaging

Three different high pressure units were used. Characteristics of these units and the target p/T/t parameter combinations of experiments performed in each unit are summarized in **Table 1**.

**Table 1:** Summary of characteristics of the three high pressure units used in this study, target p/T/t parameter combinations of experiments performed in each of the units, *C. botulinum* type E strain investigated, and determined parameters.

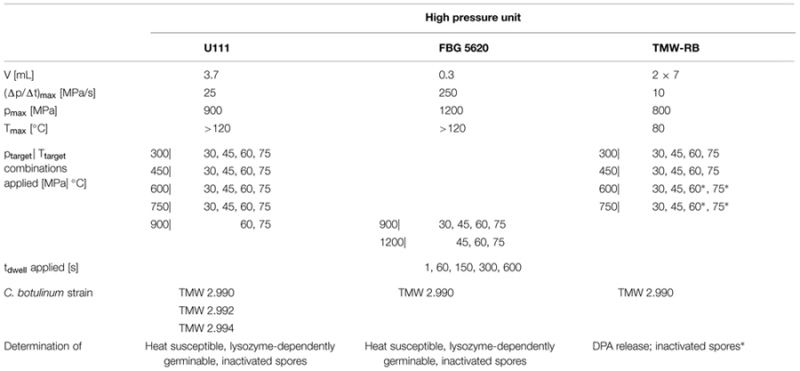

Two of the high pressure units, which were previously employed in HPT spore inactivation studies under isothermal conditions, were used to determine spore inactivation kinetics (1–600 s dwell time), i.e., unit U111 [Unipress, Poland; previously used by (Reineke et al., 2011)] and FBG 5620 [Mini Foodlab FBG 5620; Stansted Fluid Power Ltd, UK; previously used by Mathys (2008), Reineke et al. (2008), Mathys et al. (2009)]. A third dual vessel high pressure unit, TMW-RB [Knam Schneidetechnik, Germany; previously used for *C. botulinum* type E spore inactivation studies (Lenz and Vogel, 2014)], was used for experiments investigating the HPT-mediated DPA release from spores.

For experiments in unit U111, spore suspensions (200 μl) were filled in shrink tubes (DERAY^®^ – I 3000, *d*_i_ = 3.2 mm; DSGCanusa, Germany), which were hermetically sealed using a soldering iron avoiding both large air bubbles and the contact between hot matter and sample. Up to four shrink tubes were treated simultaneously in one cryovail filled with IPB avoiding the inclusion of air (1.8 mL Nunc^TM^ Cryotube, round bottom, internal thread; Thermo Fisher Scientific Inc., USA). For experiments in unit TMW-RB, cryovials were filled completely with spore suspensions and up to three vials were treated simultaneously. For experiments in unit FBG 5620, spore suspensions (75 μl) were sealed in small shrink tubes (DERAY^®^-KYF190, *d*_i_ = 1.2 mm; DSGCanusa, Germany), which were treated individually. All samples were kept on ice until treated.

### Determination of Inactivation Kinetics

#### HPT Treatments

Unit U111 (Reineke et al., 2011) was used for target pressure levels of 300–750 MPa combined with target temperatures of 30–75°C and treatments at 900 MPa/60 and 75°C (**Table 1**). Cryovails contained four shrink tubes with spore suspensions (2 × strain TMW 2.990, 1 × 2.992, 1 × 2.994). The temperature was monitored using a thermocouple located in the center of a cryovail containing the same buffer solution in which spores were suspended (IPB). Isothermal dwell times were realized immersing the pressure vessel into a temperature controlled silicon oil bath (immersion thermostat CC2-E with SilOil M40.165.10; Huber GmbH, Germany) and adjusting the pressure build-up start temperature individually for every target pressure and temperature used (Reineke et al., 2011). Process parameters were recorded in 0.5 s intervals. Experiments were conducted in duplicate using independently grown spore crops from all three strains.

Unit FBG 5620 was used for experiments at 900 MPa/30 – 75°C and at 1200 MPa/45–75°C (**Table 1**). Temperature control via a heating/cooling block allowed for realizing almost ideal adiabatic pressure build-up phases and isothermal dwell times at such high pressure levels combined with low target temperatures (Reineke et al., 2008; Mathys et al., 2009). Process parameters were recorded every 0.39 s. Experiments were conducted in duplicate with two independently grown spore crops from strain TMW 2.990.

Dwell time in both units started when a pressure level 50 MPa below the target pressure was exceeded. Due to the fast compression and related to the accuracy of the ramp control this measure increased the precision of pressure-/temperature-profiles achieved and eliminated the possibility of an unwanted prolongation of the dwell time in case the actual target pressure was not exceeded at the end of pressure ramp. Due to its suitable phase behavior within the p/T parameter range tested (Reineke et al., 2008), Bis(2-ethylhexyl) sebacate (Nr. 84822; Sigma-Aldrich, USA) was used as pressure transferring liquid (PTL).

**FIGURE 1:**
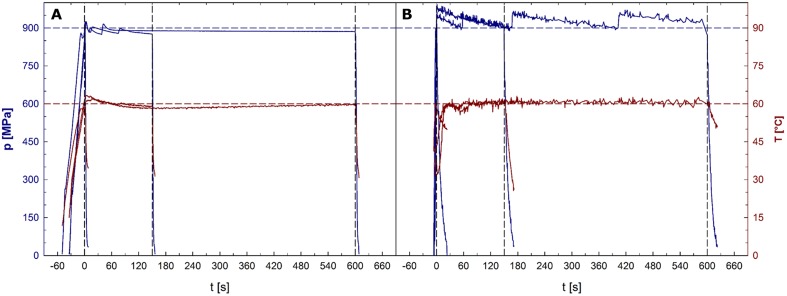
**Pressure (upper plots, left *y*-axis) and sample temperature (lower plots, right *y*-axis) profiles in the high pressure units U111 **(A)** and FBG 5620 (B).** Horizontal dashed lines indicate the target pressure of 900 MPa (upper line) and the target temperature of 60°C (lower line). Time point 0 represents the start of the dwell time. Vertical dashed lines indicate ends of dwell times after 1, 150, and 600 s.

Average pressure build-up and release rates were 25 and 132 MPa/s for unit U111 and 212 and 69 MPa/s for unit FBG 5620. In unit U111, the average pressure levels held during the dwell times were 8 MPa lower than the target pressure. Average sample temperatures reached during the dwell times of treatments with a target temperature of 30, 45, 60, and 75°C were 30.3, 44.7, 59.3, and 74.0°C, respectively. In unit FBG 5620, the average pressure levels held during the dwell times were 23 MPa higher than the target pressure. Average temperatures reached during the dwell times of treatments with a target temperature of 30, 45, 60, and 75°C were 29.1, 44.2, 59.7, and 75.1°C, respectively. Pressure and temperature profiles recorded during 900 MPa/60°C treatments in both units are depicted in **Figures 1A** (U111) and **1B** (FBG 5620).

#### Thermal Post-Treatments

One of the duplicate TMW 2.990 samples per spore crop was subjected to a second, thermal treatment after the HPT treatments. Shrink tubes were incubated 1 h at room temperature followed by 20 min at 60°C in a water bath. Samples were kept on ice after treatments.

#### Determination of Colony Forming Units

Spore inactivation was evaluated via the determination of colony forming units (CFUs). Minimum two appropriate dilutions (dilution medium S+) were plated in triplicate in TPYC agar (15 g/L agar–agar) pour plates with and without 10 μg/mL hen egg white lysozyme (minimum 100,000 u/mg, Serva, Heidelberg, Germany). CFUs were counted after anaerobic incubation at 28°C for minnimum 7 days.

### Determination of Released Dipicolinic Acid (DPA) after Pressure Treatments

#### Pressure Treatments

**FIGURE 2:**
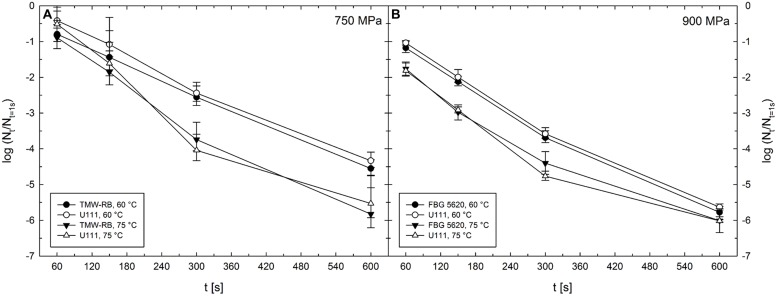
**Mean inactivation levels of *Clostridium botulinum* TMW 2.990 spores after 60–600 s HPT treatments [log (N_t_/N_1s_)] in different high pressure units. (A)** Inactivation after treatments in units TMW-RB (solid symbols) and U111 (open symbols) at a target pressure of 750 MPa and target temperatures of 60 (circles) and 75°C (triangles). **(B)** Inactivation after treatments in units FBG 5620 (solid symbols) and U111 (open symbols) at a target pressure of 900 MPa and target temperatures of 60 (circles) and 75°C (triangles). Error bars indicate SD from two (unit U111 and FBG 5620) or three (unit TMW-RB) independent experiments.

Unit TMW-RB was used for experiments to determine the HPT-mediated DPA release from *C. botulinum* TMW 2.990 spores after pressure treatments at 300–750 MPa combined with target temperatures of 30–75°C (**Table 1**). Temperature inside the pressure vessels was controlled via thermostating vessel jackets connected to an external refrigerated/heating circulator (FC 600; JULABO, Germany). The design of this unit did not allow for monitoring the actual sample temperature during HPT treatments. Thus, individual target pressure-/target temperature-dependent starting temperatures were determined empirically recording temperature profiles in the center of a dummy cryovial containing IPB. For the actual experiments, an external thermocouple was used to monitor the temperature in the center of cryovials containing IPB and shrink tubes. Ice-cold cryovials were transferred to the pre-heated pressure vessel filled with PTL. Short before (time point extrapolated from dummy test runs) the determined starting temperature was reached, the external thermocouple was removed, the lid closed, and the pressure ramp started. To be able to compare spore inactivation levels after treatments conducted using different high pressure units, spore counts after 750 MPa treatments at 60 and 75°C were also determined after treatments in unit TMW-RB (see **Figure 2**).

#### Determination of Dipicolinic Acid (DPA) Release

Similar to previous studies (Margosch et al., 2004a; Reineke et al., 2013b), high-performance liquid chromatography (HPLC) was used to determine the relative amount of DPA released from pressure treated spores. In accordance with the method used by Fichtel et al. (2007b; except for a post-column complexation), a Dionex Ultimate 3000 HPLC system (autosampler, pump; Dionex, USA) together with a Themostatted Column Compartment (TCC-100; Dionex) equipped with a Phenomenex Gemini C18 column (150 mm length, 4.6 mm *d*_i_; Phenomenex, Germany; column temperature: 20°C) was used. The column was protected with a Phenomenex Gemini C18 guard column (4 mm × 3 mm). Sodium bisulphate buffer solution (50 mM NaSO_4_; 233714, ReagentPlus^®^, 99%; Sigma-Aldrich, USA) adjusted to pH 1.2 with sulphuric acid (H_2_SO_4_, X994.1, Rotipuran^®^ 95–98%, Carl Roth, Germany) filtered through PVDF membrane disk filters (pore size 0.22 μm; 1201107; Berrytec, Germany) and degassed by ultrasonication was used as mobile phase. Isocratic elution was used for standard runs (20% methanol; 7342.1, Rotisolv^®^ HPLC gradient grade, Carl Roth; degassed with helium). A gradient program (20–40% methanol) was used for sample runs. Flow rate was set to 2 mL min^-1^. An Ultimate 3000 variable wavelength detector (Dionex) was used to measure DPA absorbance at 275 nm.

Dipicolinic acid concentrations in the surrounding medium of untreated, pressure treated, and autoclaved *C. botulinum* TMW 2.990 spores were determined. Additionally, the total amount of DPA detectable in autoclaved *C. botulinum* TMW 2.992 and TMW 2.994 spore samples was analyzed. Untreated and high pressure treated *C. botulinum* TMW 2.990 samples were kept at room temperature for 2 h after a treatment and stored at -80°C prior to HPLC analysis. For analysis, samples were filtered through cellulose acetate syringe filters (pore size 0.2 μm; F2500-16; Thermo Fisher Scientific, USA), diluted 1:1 with NaSO_4_/H_2_SO_4_ (pH 1.2), adjusted to pH 1.2 with H_2_SO_4_, and filtered again. To extract all available DPA, spore suspension samples of each batch were placed in 15 mL polypropylene screw cap tubes (62.554.502; Sarstedt, Germany) and autoclaved (208 kPa, 121.1°C) for 30 min (Pellegrino et al., 2002) followed by 1:1 dilution, H_2_SO_4_ addition, and filtration. Samples (200 μL) were placed in polypropylene HPLC vials (250 μL; 6820.0029; Thermo Fischer Scientific). Twenty microliter sample were injected per run. The use of glassware was avoided to minimize possible adsorption effects of DPA to surfaces (Fichtel et al., 2007b). 2,6-pyridinedicarboxylic acid (DPA; 2321-10G-F; Sigma-Aldrich, USA) was used as standard. Pressure-induced DPA release was calculated relative [%] to the total DPA content (autoclaved samples; Margosch et al., 2004a; Reineke et al., 2013b). Experiments were conducted independently in triplicate.

#### Calculation of Isoeffect Curves

For the calculation of isoeffect curves of heat susceptible, lysozyme activated, and inactivated spores after HPT treatments under isothermal–isobaric conditions, mean spore inactivation levels at four kinetic points (60, 150, 300, and 600 s) were considered in comparison with inactivation levels occurring during compression/decompression (1 s treatments), i.e., excluding effects under non-isobaric and non-isothermal conditions. Kinetic modeling was basically done as described before (Reineke et al., 2012, 2013b). Since no shoulder formation was observed for the inactivation kinetics of *C. botulinum* type E spores, an n^th^ order reaction model (Equation 1, Kessler, 2002) was used where the number of surviving spores (N) depends on the decrease in survivors with time (dN/dt), the corresponding rate constant (k), and the reaction order (n).

$$\frac{dN}{dt}=-k\cdot N^{n}$$

To derive isoeffect lines for pressure–temperature diagrams, kinetic analysis of the experimental inactivation data was done. The rate constants were regressively obtained (TableCurve 3D, SPSS Inc., USA) by fitting the inactivation results with n^th^-order kinetics (TableCurve 2D, SPSS Inc., USA). For n^th^-order decay reactions, Equation 1 was integrated:

$$\frac{N}{N_{0}}={\left[1+(n-1)\cdot k\cdot t\cdot N_{0}^{\left(n-1\right)}\right]}^{\frac{1}{1-n}}$$

and logarithmized:

$$\log_{10}\left(\frac{N}{N_{0}}\right)={1og}_{10}{\left[1+(n-1)\cdot k\cdot t\cdot N_{0}^{\left(n-1\right)}\right]}^{\frac{1}{1-n}}$$

To identify the reaction order, all individual kinetics were fit over a range of reaction orders (*n* = 1.0–1.7). The minimal cumulative SE (Σ SD) identified the optimal reaction order (TableCurve 2D, SPSS Inc., USA). After the identification of the reaction order (n), the rate constants (k) were obtained regressively (TableCurve 2D, SPSS Inc., USA). To get a functional relationship of the rate constant with pressure and temperature dependence k(p,T), empirical equations have often been suggested (Ardia, 2004; Ardia et al., 2004; Margosch et al., 2006). Using a Taylor series expansion up to third order terms,

$$\ln(k)=a+b\cdot p+c\cdot T+d\cdot p^{2}+e\cdot T^{2}+f\cdot p\cdot T+g\cdot p^{3}+h\cdot T^{3}$$

the rate constant k(p,T) could be calculated. To solve the functional relationship between pressure and temperature, k was replaced with k(p,T) and the reduction rate (N/N_0_) and time (t) were set as constants. The isoeffect lines were calculated with MathCAD 15; Mathsoft Engineering & Education, USA). The calculated parameters for the Taylor series expansion and its confidence intervals are given in Table 1 of the supplemental material.

Since the DPA release data were differently structured, an alternative modeling approach was used for the calculation of isoeffect lines for the release of DPA from spores after HPT treatments. In contrast to the inactivation isoeffect lines, a significant DPA amount was already released during pressure build-up, which made it necessary to consider both effects occurring under static conditions (isothermal, isobaric dwell times) as well as non-isobaric and non-isothermal conditions (during pressure build-up) in the model. Hence a Weibullian power law:

$$\log_{10}\left(\frac{N_{t}}{N_{0}}\right)=-b\cdot t^{n}$$

was used to calculate the functional relationship of the scale (b) and shape parameter (n) in dependence of pressure and temperature.

By applying the Weibullian power law, a non-linear regression fit was done for each individual DPA release kinetic and the scale and shape parameter were determined. To get a functional relationship of the scale parameter b with pressure and temperature, it was assumed that the shape parameter n is constant with varying pressure and temperature (Van Boekel, 2009). Therefore, the average of all values for the shape parameter was calculated (0.23) and the non-linear regression fit of each inactivation kinetic was repeated with this fixed value for the shape parameter.

To get a functional relationship of the scale parameter b with pressure and temperature the same method as described above for the n^th^ order reaction model was used.

## Results

### Spore Inactivation in Different High Pressure Units

To be able to compare the results from experiments under isothermal–isobaric conditions conducted in the high pressure units U111 and FBG 5620 and the results obtained for the HPT-mediated release of DPA from spores conducted in unit TMW-RB, it is essential that treatments with identical target parameters (p/T/t) in the different units exert comparable effects on spores. Spore inactivation levels obtained after treatments in the different high pressure units excluding effects under non-isobaric and non-isothermal conditions [log (N_t_/N_1s_)] are shown in **Figure 2**.

The absence of significant differences between inactivation results obtained using the three different high pressure units demonstrates that stress intensities at identical target parameters are comparable independently of the unit used. Most importantly this indicates that the empirical adjustment of starting temperatures and the experimental setting of experiments in unit TMW-RB (used to examine the HPT-mediated DPA release) were suitable to obtain similar spore inactivation levels compared with the other two units, where the actual sample temperatures were precisely controlled.

### Inactivation Kinetics and DPA Release

**FIGURE 3:**
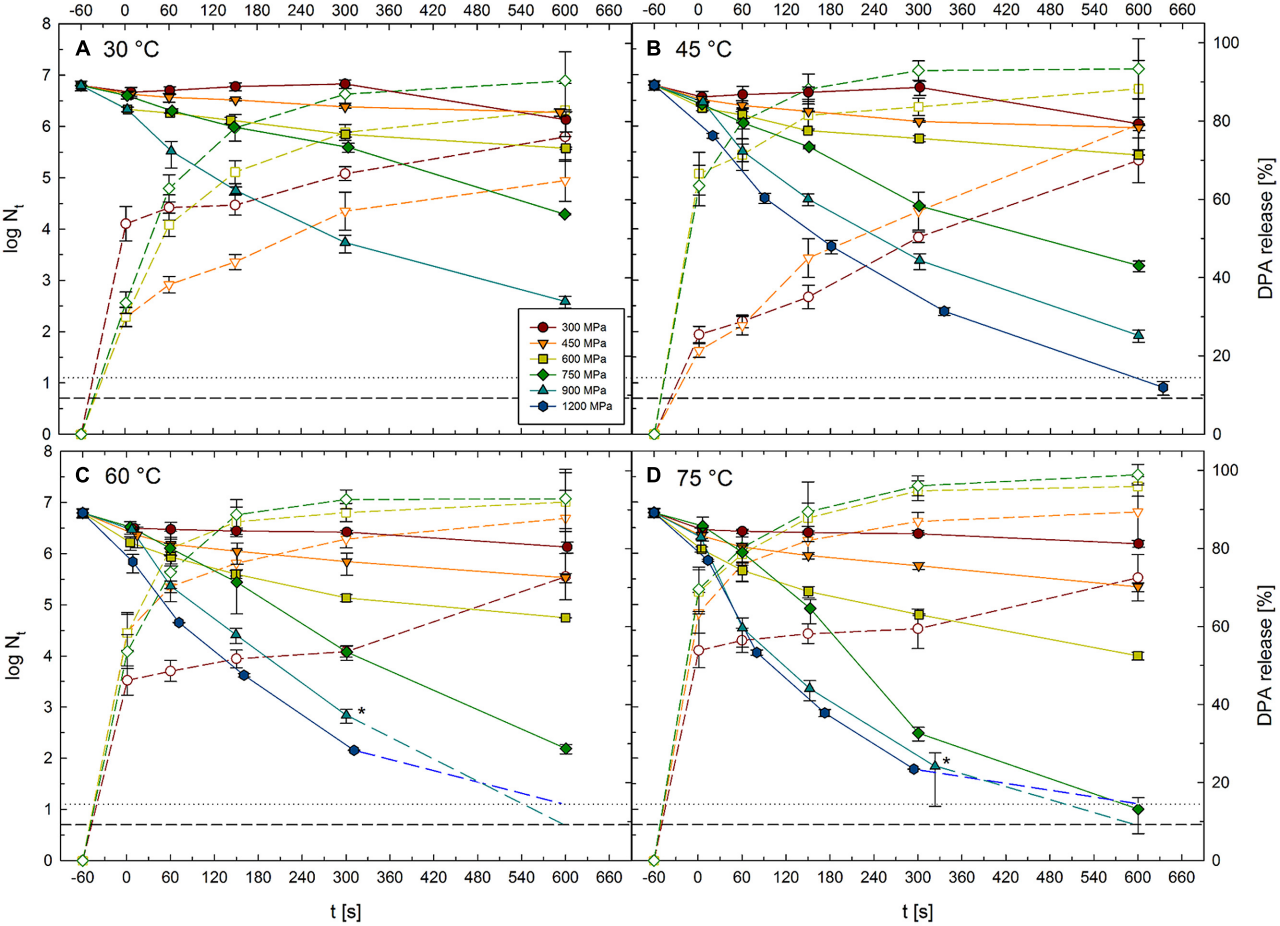
**Kinetics of surviving *C. botulinum* TMW 2.990 spores and accompanying release of DPA after HPT treatments at 300–1200 MPa combined with **(A)** 30°C, **(B)** 45°C, **(C)** 60°C, and **(D)** 75°C for 1–600 s.** Solid lines and symbols indicate viable spore counts (left *y*-axis) after high pressure thermal (HPT) treatments. Short dashed lines and open symbols indicate DPA release (right *y*-axis). Colors and symbol shapes indicate the pressure intensity of a treatment, i.e., 300 (red circles), 450 (orange downward triangles), 600 (yellow squares), 750 (green diamonds), 900 (cyan upward triangles), and 1200 MPa (blue hexagons). Inactivation by 1200 MPa/30°C and DPA release after 900 and 1200 MPa treatments was not determined and is therefore not depicted. Horizontal lines at the bottom of each graph represent detection limits for viable spore counts after treatments in unit FBG 5620 (dotted line, 1.125 log) and unit U111 (short dashed line, 0.7 log). For spore counts below the detection limit, plots are extended toward this limit with medium dashed lines. Asterisks mark mean values for spore counts from the units FBG 5620 and U111. Error bars indicate SD from two (viable spore counts, units U111 and FBG 5620) or three (DPA release, unit TMW-RB) independent experiments.

Viable spore counts of *C. botulinum* TMW 2.990 before and after HPT treatments at 300–1200 MPa combined with temperatures of 30–75°C for dwell times of 1–600 s (high pressure units U111 and FBP 5620; **Table 1**) are shown in **Figure 3**. Kinetics for 1200 MPa at 30°C are missing, because it was not possible to realize isothermal treatment conditions applying such high pressure levels combined with low target temperatures. This was related to the fact that the adiabatic heat of compression, i.e., the temperature raise in the sample during pressure build-up, exceeds the target temperature (30°C) by far. Additionally, water in the sample would have been in the frozen state (ice VI) at 1200 MPa/30°C, which would have largely influenced inactivation results. **Figure 3** also contains data for the relative amount of DPA released from spores in response to 300–750 MPa treatments at 30–75°C for dwell times of 1–600 s (high pressure unit TMW-RB).

Spore inactivation generally increased with increasing treatment intensity and duration. Thus, data presented here do not point toward the existence of any p/T zones where inactivation is significantly retarded. Pressure levels of 300 and 450 MPa had a weak effect on spore counts regardless of the temperature at which the treatments were conducted (30–75°C). When only isothermal–isobaric dwell times are considered [log (N_t_/N_1s_)], spore inactivation by such treatments did not exceed 1 log cycle, and maximum inactivation levels reached after treatments at 300 and 450 MPa were 0.6 log (at 30 °C, 600 s) and 1 log (at 45°C, 600 s), respectively. Spore inactivation was markedly increased when pressures above 600 MPa were used. Maximum log inactivation values at 600 and 750 MPa target pressure were 2.1 and 5.6, respectively (reached at 75°C, 600 s). Maximum log inactivation at 900 or 1200 MPa were also found in combination with 75°C (600 s), which in both cases resulted in a complete inactivation of the spore population (>6 log). The absolute maximum of inactivation reached at 30°C was 3.8 log (900 MPa, 600 s; note: 1200 MPa/30°C experiments not conducted). The highest inactivation levels reached at target temperatures of 45, 60, and 75°C were 4.9, 5.8, and >6 log, respectively (all in combination with 1200 MPa, 600 s).

Similar to the inactivation levels, DPA release increased with treatment intensity and dwell time. As an exception, the maximum relative amount of DPA released from spores after treatments at 300 MPa was 76% after 600 s at 30°C, which was slightly higher than the amounts released after 300 MPa treatments at 45, 60, or 75°C. Maximum relative DPA amounts released after 450, 600, and 750 MPa were 92, 96, and 99% each after 600 s treatments at 75°C. Comparing DPA release after treatments at identical target temperatures, maximum DPA release reached after 30, 45, 60, and 75°C treatments were 90, 93, 93, and 99%, respectively (all after 600 s at 750 MPa). Fastest DPA release occurred at 75°C where 52, 64, 67, and 70% of the total DPA content of spores was released in response to a short (1 s) pressure pulse at 300, 450, 600, and 750 MPa, respectively. However, short dwell times at 300 MPa/30°C were also relatively effective in triggering the release of DPA (54% after pressure build-up), which exceeded the release after 1 s treatments at 300 MPa/45°C (25%), 300 MPa/60°C (46%), 450 MPa/30°C (30%), 600 MPa/30°C (30%), and 750 MPa/30°C (34%) by far.

### Heat Susceptible Fraction after HPT Treatments

Heat treatments (60°C) subsequent to the HPT treatments were employed to give an estimate on the number of spores that lost their high heat resistance in response to HPT but were not inactivated during such treatments. This fraction can contain (i) spores that germinated during or within 2 h after a HPT treatment following the physiological pathway as well as (ii) spores that somehow lost their heat resistance (commonly associated with DPA release and core rehydration) or (iii) spores that are sublethally damaged (e.g., in molecules essential for outgrowth) and for which a heat treatment at 60°C becomes lethal.

**FIGURE 4:**
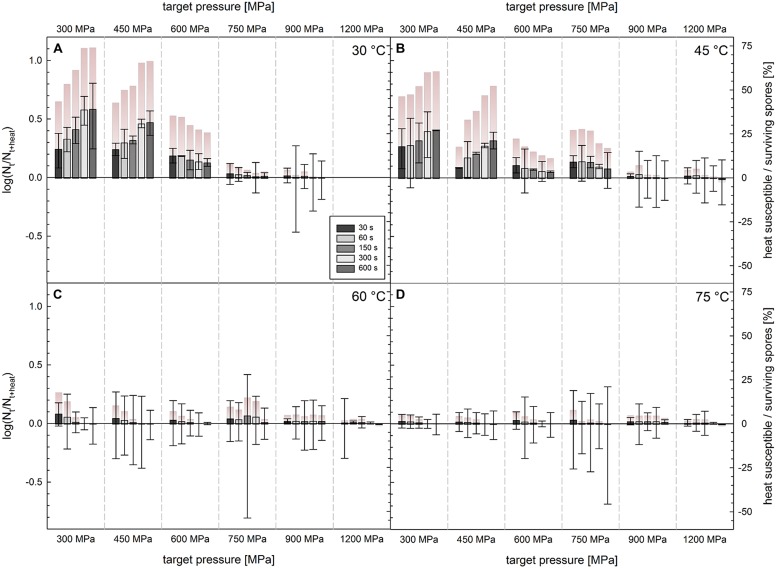
**Difference in survivors of *C. botulinum* TMW 2.990 spores after HPT treatments with and without a subsequent heating step at 60°C [log (N_t_/N_t__+__heat_); left y-axis].** HPT treatments at **(A)** 30°C, **(B)** 45°C, **(C)** 60°C, and **(D)** 75°C. Columns (x-axis) indicate different target pressures (30°C/1200 MPa experiments no conducted). Different color intensities of bars indicate different dwell times. Positive and negative values correspond to lower and higher numbers of CFU, respectively, after the additional heat treatment. Error bars indicate the square root of the sum of square of errors for HPT treated and HPT and heat treated samples derived from two independent experiments. Color gradient bars indicate the number of heat susceptible spores (or cells) relative to the number of spores (or cells) surviving a HPT treatment ((N_t_–N_t__+__heat_)/N_t_ × 100 [%]; right y-axis).

The difference in the number of viable spores (CFU/mL) detected after HPT treatments and that after a subsequent additional heat treatment at 60°C [log(N_t_/N_t__+__heat_)] was generally low and did not exceed 0.59 log observed after 300 MPa/30°C/600 s treatments, which corresponds to 74% of the spore population surviving this HPT process (**Figure 4**). Very small but significant differences were also found after treatments at 300 MPa/45°C, 450 MPa/30°C, and 450 MPa/45°C, i.e., up to 0.41, 0.47, and 0.25 log for a dwell time of 600 s, respectively. This corresponds to 61, 66, and 52% of the surviving population without applying a second heat treatment. After treatments at the remaining pressure/temperature combinations (>450 MPa/>45°C), plate count results were virtually identical, no matter if HPT treatments were followed by a second thermal treatment or not.

### The Effect of Lysozyme in the Recovery Medium

**FIGURE 5:**
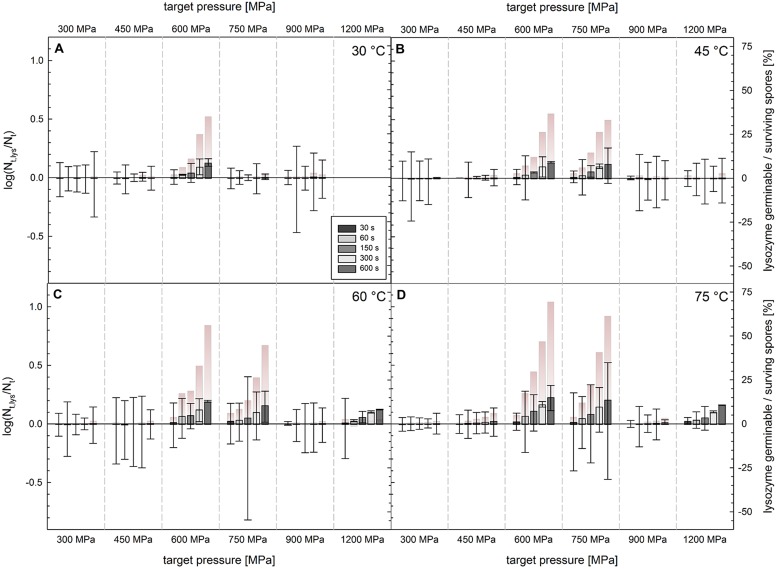
**Difference in survivors of *C. botulinum* TMW 2.990 spores after HPT treatments enumerated on plate with and without lysozyme [log(N_t__+__lysozyme_/N_t_); left *y*-axis].** HPT treatments at **(A)** 30°C, **(B)** 45°C, **(C)** 60°C, and **(D)** 75°C. Columns (*x*-axis) indicate different target pressures (30°C/1200 MPa experiments no conducted). Different color intensities of bars indicate different dwell times. Positive and negative values correspond to higher and lower numbers of CFU on plates containing lysozyme. Error bars indicate the square root of the sum of square of errors for HPT treated samples plated on plates with and without lysozyme. Color gradient bars indicate the number of lysozyme-dependently germinable spores relative to the number of spores surviving a HPT treatment ((N_t__+__lysozyme_/N_t_)/N_t_ × 100 [%]; right *y*-axis).

Plate counts with and without lysozyme in the recovery medium were carried out to detect spores with both defects in their cortex lytic machinery and coat layers permitting access of lysozyme to the peptidoglycan layer underneath it. Besides of subtle but significant differences that appeared to exist when spores were treated at 600 and 750 MPa, which tended to increase with increasing treatment temperature and dwell time, CFU/mL found in pour plates with and without lysozyme were generally not significantly different from each other (**Figure 5**). The maximum difference observed was 0.23 log [log(N_t__+__lysozyme_/N_t_)] after treatments at 600 MPa/75°C for 600 s corresponding to 69% of the surviving spore fraction detected in plates without lysozyme [(N_t__+__lysozyme_/N_t_)/N_t_ × 100]. Slightly lower lysozyme-dependent log differences in spore counts were found for samples treated at 750 MPa/75°C/600 s (0.21 log, 61%), 600 MPa/60°C/600 s (0.19 log, 56%), and 600 MPa/75°C/600 s (0.16 log, 45%).

### Isoeffect Curves

**FIGURE 6:**
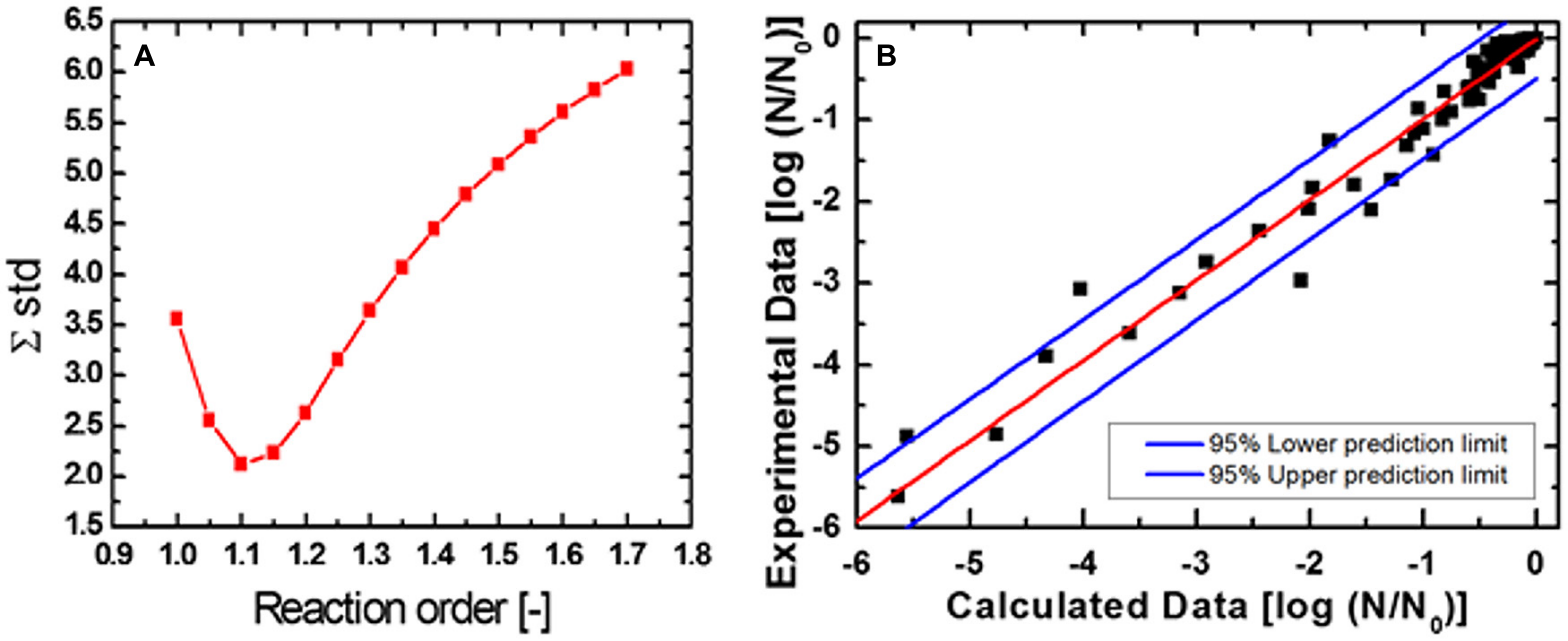
**Sum square error plotted against reaction order **(A)** and comparison between calculated and experimental data **(B)** for the inactivation of *C. botulinum* TMW 2.990 spores**.

To facilitate the comparison of data obtained for *C. botulinum* TMW 2.990 spore inactivation, the heat susceptible fraction after HPT treatments, and the effect of the presence of lysozyme in the recovery medium, 1 log and 3 log isoeffect lines were calculated as it has been described previously (Reineke et al., 2012). **Figure 6** shows the empirical determination of the reaction order (*n* = 1.1; **Figure 6A**) and the comparison between calculated and experimental data (**Figure 6B**), which resulted in a good fit (adjusted *R*^2^ = 0.97 for spore inactivation). Random residual distribution indicates the absence of a large heteroscedastic error.

**FIGURE 7:**
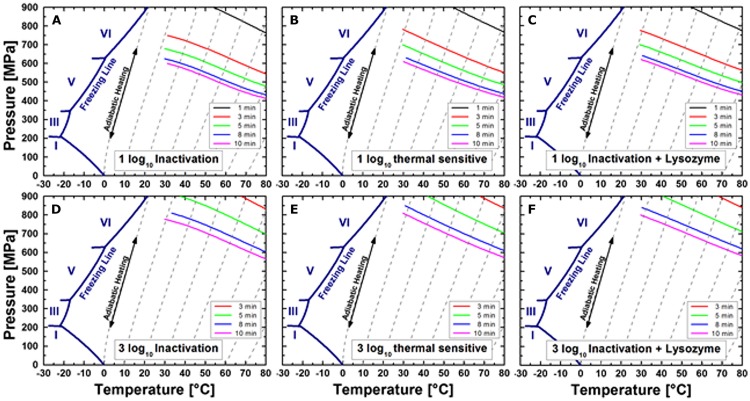
**Isoeffect lines for a 1 log **(A–C)** and 3 log **(D–F)** inactivation of *C. botulinum* TMW 2.990 determined directly after HPT treatments in standard TPYC plates **(A,D)**, including a second heat treatment at 60°C **(B,E)**, and plated in standard TPYC plates containing lysozyme.** Different colors indicate different dwell times under isothermal–isobaric conditions, i.e., 1 min (black), 3 min (red), 5 min (green), 8 min (blue), and 10 min (pink).

**Figure 7** shows the calculated isoeffect lines for a 1 log and 3 log inactivation after 3, 5, 8, and 10 min isothermal–isobaric dwell time determined directly after HPT treatments without lysozyme in the recovery medium (A and D), with an additional heating step after the HPT process (B and E), and with lysozyme in the recovery medium (C and F).

In accordance with the small differences between the viable spore counts of samples analyzed directly after HPT treatment and determined after a second heat treatment (**Figure 4**) or when lysozyme was added to the recovery medium (**Figure 5**), calculated isoeffect lines shown in **Figure 7** display almost identical shapes regardless of an additional heat treatment or the recovery conditions. This illustrates the absence of large portions of heat susceptible or lysozyme-dependently germinable spores after HPT treatments described above.

**FIGURE 8:**
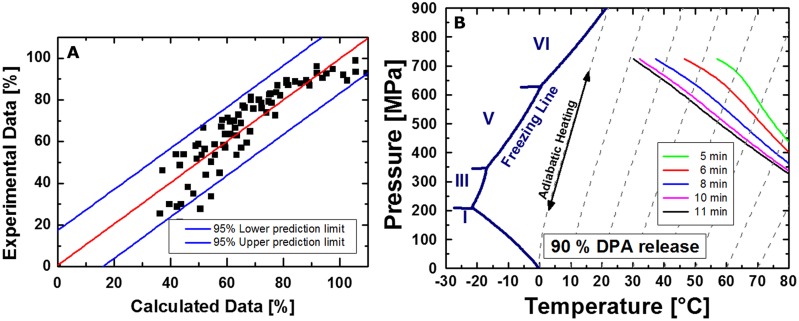
**Comparison between calculated and experimental data **(A)** and isoeffect lines **(B)** for a 90% DPA release from HPT treated *C. botulinum* TMW 2.990 spores determined 2 h after the respective treatment.** Data for the calculation are derived from experiments in unit TMW-RB at 300–750 MPa and 30–75°C including DPA release data occurring during pressure-come up. Different colors of isoeffect lines indicate different total process time (including 1 min come-up), i.e., after 5 min total process time (green), 6 min (=5 min dwell + 1 min come-up; red), 8 min (blue), 10 min (pink), and 11 min (= 10 min dwell + 1 min come-up; black).

**Figure 8** shows the comparison between calculated and experimental data (**Figure 8A**) for the release of DPA from *C. botulinum* TMW 2.990 spores after HPT treatments, which resulted in an adequate fit (adjusted R^*2*^ = 0.93). However, the fact that the residuals are not entirely stochastically distributed indicates the possible presence of a heteroscedastic error. This is related to the fact that the model assumes isothermal–isobaric conditions to enable the calculation of isoeffect lines. However, due to the considerable DPA amount already released during pressure build-up, non-isobaric and non-isothermal conditions had to be included in the model. **Figure 8B** depicts calculated isoeffect lines for a 90% release of DPA for different dwell times dependent on the target pressure und process temperature. To facilitate the comparison with inactivation isoeffect lines (**Figure 7**), lines for 6 min corresponding to 5 min dwell + 1 min come-up (red) and 11 min corresponding to 10 min dwell + 1 min come-up (black) are included. Similar to the results for inactivation, high pressures levels at low temperatures or low pressure levels at high temperatures are required to provoke the released of the same amount of DPA from spores. This dependency appears to be almost linear for longer dwell times.

### Strain-Specific HPT Resistance and DPA Content

In addition to *C. botulinum* type E strain TMW 2.990, inactivation levels of strains TMW 2.992 and TMW 2.994 after isothermal–isobaric HPT treatments at 300–750 MPa and 30–75°C for 1–600 s were determined. Both strains were generally less resistant to HPT treatments than strain TMW 2.990. Considering all investigated p/T/t conditions, average differences in log inactivation occurring during isothermal–isobaric dwell times (Δlog N_1s_/N_t_) between strain TMW 2.990 and TMW 2.992 and between TMW 2.990 and TMW 2.994 were 0.3 and 0.4 log cycles, respectively. Despite of the usual similar shapes of inactivation curves, the extent of differences appeared to be process intensity-dependent. With a few exceptions, strain-specific differences in inactivation levels tended to increase with increasing pressure, temperature, and dwell time.

**FIGURE 9:**
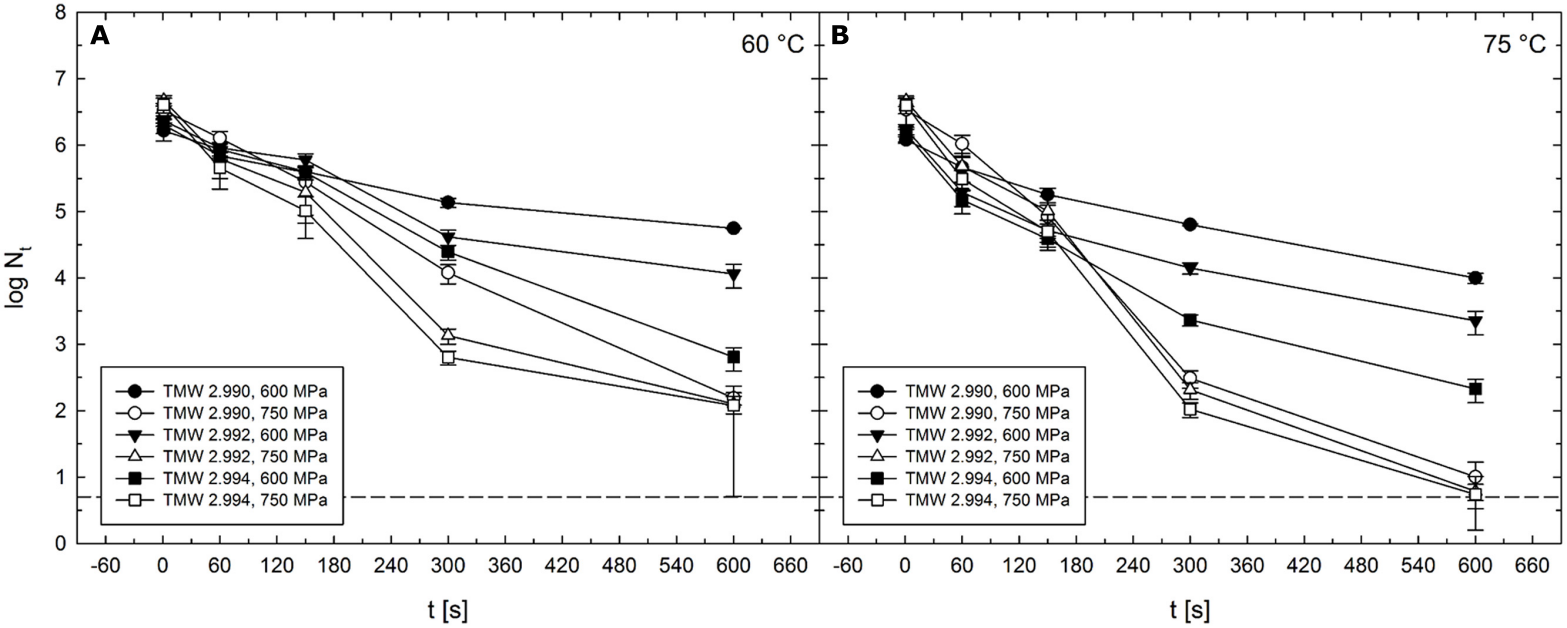
**Mean levels of *C. botulinum* type E strains TMW 2.990 (circles), TMW 2.992 (triangles), and TMW 2.994 (squares) spores surviving 1–600 s HPT treatments (log N_t_) in unit U111 at target pressure of 600 MPa (solid symbols) and 750 MPa (open symbols). (A)** In combination with 60°C and **(B)** in combination with 75°C. The dashed line indicates the detection limit. Error bars indicate SD from two independent experiments.

Largest strain-specific differences in inactivation levels were observed after HPT treatments at 600–750 MPa at 60°C (**Figure 9A**) or 75°C (**Figure 9B**). Absolute maxima of differences between strain TMW 2.990 and the other strains were 1.1 log for TMW 2.992 (750 MPa/60°C/300 s) and 2 log for TMW 2.994 (600 MPa/60°C/600 s) (**Figure 9**). Second largest differences between strain TMW 2.990 and TMW 2.992 were 0.8 log (600 MPa/60 and 75°C/600 s). Second largest differences between strain TMW 2.990 and TMW 2.994 were 1.8 (600 MPa/75°C/600 s) and 1.3 log (750 MPa/60 °C/300 s). Thus, with the exception of treatments at 750 MPa/75°C, strain-dependent differences appeared to progressively increase with increasing pressure and/or temperature reaching maximum levels when pressure levels above 600 MPa and process temperatures above 60°C were used (**Figure 9**).

This trend can also be observed for average differences occurring after all isothermal–isobaric dwell times (60 and 600 s) in a specific pressure/temperature range. Average differences in inactivation levels between the strains TMW 2.990 and TMW 2.992 ranged between 0.1 and 0.3 log when spores from both strains were subjected to HPT treatments at 300 MPa/45–75°C, 450 MPa/45°C, 600 MPa/30–45°C, and 750 MPa/45°C. Larger differences between the inactivation levels, i.e., in a range between 0.3 and 0.7 log, were found after HPT treatments at 450 MPa/60–75°C, 600 MPa/60–75°C, and 750 MPa/30, 60, and 75°C.

Average differences in inactivation levels between the strains TMW 2.990 and TMW 2.994 occurring during isothermal–isobaric dwell times of 60–600 s ranged between 0.1 and 0.3 log cycles after HPT treatments at 300 MPa/45–75°C, 600 MPa/30°C, and 750 MPa/45°C. A range between 0.3 and 0.7 log was found after HPT treatments at 450 MPa/60–75°C, 600 MPa/45°C, and 750 MPa/30, 60, and 75°C. Between 0.8–1.2 log were detected after 600 MPa/60 and 75°C treatments. In contrast, no significant differences were observed between any of the strains after HPT treatments at 300 or 450 MPa at 30°C (data not shown).

In addition to comparing inactivation levels, data obtained here allows for a comparison of strains regarding their total amount of DPA detectable in autoclaved spore samples (HPLC analysis). The determination of total spore counts using a counting chamber (0.01 mm depth) and phase contrast microscopy enabled the calculation of DPA concentration per spore. Three different spore crops were analyzed and average DPA levels per spore were 155 ± 23, 132 ± 9, and 72 ± 14 amol (=10^-18^ mol) for *C. botulinum* strains TMW 2.990, TMW 2.992, and TMW 2.994, respectively. This corresponds to an average of 9.3 ± 1.4 × 10^7^, 8.0 ± 0.5 × 10^7^, and 4.3 ± 0.8 × 10^7^ DPA molecules per spore.

## Discussion

### Process Control and Comparability of Results

Spore inactivation data derived under isothermal–isobaric conditions excluding effects during pressure build-up and release can be compared between different high pressure units (**Figure 2**). This is in accordance with earlier findings, where the exclusion of non-isothermal and non-isobaric conditions resulted in a perfect fit for models derived from kinetic data using different high pressure equipment (*B. subtilis* spores, Reineke et al., 2012). Provided that inactivation data are comparable between different high pressure units, it can be assumed that kinetic data for DPA release are also comparable between these units (Reineke et al., 2013b). The comparability of kinetic data is not only important for the interpretation of the results showed here but also presents a prerequisite of the direct comparability of results from different spore inactivation studies, which are usually conducted using various high pressure equipment. This becomes especially important when high process temperatures are used, which is the common case when the inactivation of bacterial endospores is investigated.

Notably, although the process temperature was controlled within a narrow range during treatments in unit FBG 5620, it cannot be excluded that pressure peaks (temporary pressure levels exceeding the target pressure) during treatments in this unit (**Figure 1**) resulted in a slight overestimation of the effectiveness of treatments, i.e., a detection of higher inactivation levels than those that might have been found if ideal isobaric conditions would have been prevalent during a treatment. This might especially be the case where the temperature can be assumed to play a minor role for inactivation (30–45°C), very high pressure levels were used (750–1200 MPa), and experiments were conducted solely in unit FBG 5620 (i.e., 750 and 900 MPa/30°C, 900 and 1200 MPa/45°C).

### Inactivation Kinetics

Inactivation of *C. botulinum* type E strain TMW 2.990 (Beluga) spores is retarded at <600 MPa/<60°C (maximum 0.7 log reduction; **Figure 3**), which is in accordance with results from previous studies for the same strain (Reddy et al., 1999; Lenz and Vogel, 2014). Although spores from non-proteolytic *C. botulinum* strains are markedly less resistant to harsh environmental conditions (physical treatments) than spores from several other species, inactivation levels achieved after treatments at low pressure/moderate temperatures are similar or lower than those reported for various other spores (e.g., *B. subtilis*, Reineke et al., 2012). Under such processing conditions, inactivation is likely to occur as a result of pressure-induced germination and loss of physiological fitness, whereas there are only reversible or minor changes in the overall spore structure (e.g., 200 MPa, 30–55°C, *Geobacillus stearothermophilus*, Georget et al., 2014). Thus, it seems reasonable that either germination is not efficiently triggered or the physiological fitness of *C. botulinum* type E spores is not impaired during low pressure/moderate temperature treatments. The former could be related to a low responsiveness of *C. botulinum* nutrient germinant receptors (nGRs) to pressure. Additionally, inner membrane properties (composition/rigidity/phase behavior) might play a role, since the pressure-induced formation of a gel phase with different packing properties and lateral organization has been proposed to contribute to the activation of nGRs (Georget et al., 2014).

An increase in target pressure and/or temperature accelerated inactivation, which is also consistent with previous findings (Reddy et al., 1999; Lenz and Vogel, 2014). When effects occurring during pressure build-up are included [log(N_0_/N_t_)], HPT inactivation levels at moderate temperatures were similar compared with those reported earlier (Reddy et al., 1999). For example, after 5 min at 827 MPa/45°C were reported to result in an ∼2.4 log inactivation of *C. botulinum* TMW 2.990 (Beluga) spores (Reddy et al., 1999), which is similar to the 2.3–3.4 log inactivation after 750–900 MPa/45°C treatments reported here. The significant inactivation by high pressure/moderate temperature treatments is in contrast to a low or no inactivation frequently reported for *Bacillus* spores (Wuytack et al., 1998; Ardia, 2004; Rajan et al., 2006; Mathys et al., 2009; Reineke et al., 2012).

At higher process temperatures (≥60°C), differences between inactivation levels determined here and those reported earlier for non-proteolytic *C. botulinum* spores (Reddy et al., 1999; Skinner et al., 2014) become apparent. For example, a 5 log inactivation after 5 min at 827 MPa/55°C (Reddy et al., 1999) clearly exceeds the 2.7–3.9 log inactivation after 750–900 MPa/60°C treatments found here. Such differences are putatively related to process temperatures exceeding the target temperature in the early phase of less precisely controlled HPT processes (adiabatic peak after pressure build-up). An increase in the process intensity accelerated inactivation resulting in an >6 log reduction [log(N_0_/N_t_)] after 1200 MPa/45°C or 750 MPa/75°C treatments and a complete inactivation of ∼6 × 10^6^ spores per mL after 10 min at 900 and 1200 MPa at 60 or 75°C (**Figure 3**).

Spore inactivation at high pressure/elevated temperature is thought to occur due to a multi-stage mechanism beginning with sublethal injury of spores by heat and pressure (Margosch et al., 2004a,b) and/or DPA release due to the inner membrane losing its barrier function (Mathys et al., 2007). In the latter case, core rehydration and the accompanying loss of heat resistance facilitates spore inactivation by moderate heat, where the pressure level plays a less important role (Margosch et al., 2004a,b). Inactivated spores were reported to be characterized by a physically compromised inner membrane (Mathys et al., 2007). Germinant receptors are thought to play no role in the HPT-mediated DPA-release triggered under such conditions (Mathys et al., 2007; Reineke et al., 2013a). Thus, this process is commonly referred to as non-physiological germination (Reineke et al., 2013a). In comparison with proteolytic *C. botulinum* strains (Margosch et al., 2004a,b) and *G. stearothermophilus* (Mathys et al., 2007) for which these steps have been described, *C. botulinum* type E spores appear to be significantly more susceptible to both heat and pressure. In contrast to earlier studies where particular p/T combinations resulted in lower spore inactivation than heat alone and a pronounced pressure-dependent tailing effect [*B. amyloliquefaciens*, 800–1200 MPa/120°C (Margosch et al., 2006); proteolytic *C. botulinum*, 700 MPa/121 °C, packaging-dependent, possibly related to inhomogeneous processing, (Patazca et al., 2013)], zones of spore stabilization were not observable for *C. botulinum* type E spores.

There were virtually no vegetative cells remaining in the purified spore suspensions. Thus, neglecting the possibility that dormant spores, which are not detected by initial plate counts but activated by a specific treatment (e.g., a sublethal heat or pressure), the reduction in viable counts after HPT treatments equals the inactivation of initially viable spores. Generally, this inactivation can occur due to damages in spore components essential for spores to develop into a vegetative, growing and doubling cell. Although such damages do not necessarily lead to the release of DPA from the spore core, DPA release (and concomitant core rehydration and loss of resistance properties) was reported to present the rate-limiting step in the inactivation of *Bacillus* spores (*B. subtilis*, Reineke et al., 2013b). However, comparable data on the HPT-mediated release of DPA from *Clostridium* spores is limited and some species-dependent differences seem to exist (Hölters et al., 1999; Margosch et al., 2004a; Hofstetter et al., 2013a), which is discussed in the following paragraphs.

### DPA Release

Similar to the inactivation levels, DPA release from *C. botulinum* type E (strain TMW 2.990) spores generally increased with increasing treatment intensity and longer dwell times. Only one exception was observed, i.e., the DPA release after 300 MPa/30°C treatments. After such treatments, DPA release occurred considerably slower compared with treatments at high pressure/high temperature but significantly faster and more effective than after moderate heat/moderate temperature treatments (**Figure 2**). Generally, HPT treatment conditions sufficient to provoke more than 2 log cycles (>99%) inactivation (minimum 600 MPa/60°C/600 s) were required to trigger the release of 90% of the spores’ total DPA content. After treatments at the highest intensity tested (750 MPa/75°C/600 s), the inactivation level reached 5.8 log cycles and 99% of the total DPA content were released. A fast DPA release from *C. botulinum* type E spores has also been reported to occur during heat processing at 75°C, where almost 60 and 90% of DPA were released after 5 and 10 min dwell time, respectively, corresponding to an ∼0.5 and 1 log inactivation [strain Vancouver Herring (VH), Grecz and Tang, 1970].

In comparison with *C. botulinum* type A, type E spores are significantly less resistant to heat and HPT treatments and appear to lose their DPA considerably faster. Type A spores were reported to release only ∼25% of their DPA after 800 MPa/80°C/600 s treatments at pH 6 (Margosch et al., 2004a). A complete release was observed only under conditions leading to an >5 log reduction (>99.999%), i.e., at 800 MPa/116°C for >1 h (∼70% release after 600 s; Margosch et al., 2004a). In contrast, a substantial DPA release from *C. beijerneckii* and *C. sporogenes* spores was shown to occur within <5 min at 600 MPa/90°C (Hofstetter et al., 2013a) and a considerable DPA release from *C. pasteurianum* spores was described after relatively mild HPT processes (Hölters et al., 1999). Albeit DPA release from *C. pasteurianum* spores appears to occur consistently slower after 300 MPa treatments and faster after 450 MPa treatments (Hölters et al., 1999), reported levels are similar to those found here for *C. botulinum* type E. For example, 300 MPa/60°C/60–600 s treatments resulted in ∼40–60% DPA release from *C. pasteurianum* (Hölters et al., 1999) and 49–73% from *C. botulinum* type E spores.

Low pressure levels can trigger a physiologic-like (similar to nutrient-induced) germination pathway, where the activation of nGRs provokes the release of over 90% of the large depot of DPA present in the core as a 1:1 chelate with divalent cations (predominantly Ca^2+^; Rode and Foster, 1966; Paidhungat and Setlow, 2000, 2001; Wang et al., 2011). Consequentially, nGR levels are the major factor determining low pressure-mediated germination rates whereas other germination related proteins play a minor role (*B. subtilis*, Doona et al., 2014). Typically, pressure levels between 80 and 100 MPa (Paidhungat et al., 2002; Torres and Velazquez, 2005; Margosch et al., 2006) and 150 MPa (Gould and Sale, 1970; Reineke et al., 2012) can efficiently trigger germination of different *Bacillus* spores in the absence of nutrients [temperature and pH-dependent, (Gould and Sale, 1970; Reineke et al., 2012)]. Possibly due to (reversible) conformation modifications of nGR domains at the spores’ inner membrane periphery (Georget et al., 2014). Though less effective, physiologic-like germination was reported to be also triggered at pressure levels of 300 up to 600 MPa (threshold pressure) at tempertures up to 50°C (*B. subtilis*, Wuytack et al., 1998; Reineke et al., 2013b).

*Bacillus* and *Clostridium* spores have considerable differences in nutrient germination requirements, which is putatively related to the different genetic architecture (Paredes-Sabja et al., 2011) and different conformation of their nGRs. Hence, it is not surprising that *Clostridium* nGRs also harbor a different responsiveness to HPT, and that HPT levels triggering nGR-mediated germination of *B. subtilis* spores do not provoke identical effects in *Clostridium* spores. Although the pressure levels at which physiologic-like germination is triggered and the effectiveness of this process may differ between spores from different species, the observed DPA release from *C. botulinum* type E even after very short dwell times is in accordance with findings for *B. subtilis* where a short pressure pulse (150 MPa, 30 s) can be sufficient to commit spores to germination (Kong et al., 2014). Notably, since only the total amount of released DPA was measured, it is not possible to define whether the DPA originates from a certain germinated spore fraction or presents a partial release from the majority of spores. However, the accelerated DPA release from *C. botulinum* type E spores after 300 MPa/30°C treatments compared with that after moderate heat/moderate temperature treatments (**Figure 2**) might be attributed to pressure-induced germination of a distinct spore fraction.

At higher pressure levels and increased process temperatures, DPA release from *C. botulinum* type E spores was significantly accelerated. Although temperatures required for a substantial DPA release after HPT treatments at high pressure/elevated temperatures are somewhat lower than those reported for other spore formers, the rapid DPA release under such conditions presents a common response among *Bacillus* (Reineke et al., 2013b) and *Clostridium* (Margosch, 2004) spores. In *Bacillus* spores, high pressure/elevated temperature treatments are thought to trigger a non-physiological DPA release, which leads to a partial core rehydration but incomplete germination (Reineke et al., 2013a) and occurs independently from the presence of functional nGRs (Black et al., 2007). Although the exact mechanism underlying this rapid DPA release remains to be elucidated, there exists some knowledge, which allows for discussing factors that might determine organism-specific differences in the non-physiological DPA release rates.

Whereas effects on the solubility of DPA seem unlikely (core DPA concentration exceeds solubility limit by far), (i) inner membrane properties and (ii) cortex lytic enzymes (CLEs) present potential factors that might play a role.

(i) Biological membranes can undergo phase transition under pressure and are generally recognized as one of the most pressure-sensitive cellular components (Winter and Jeworrek, 2009). Consequently, pore formation due to inner membrane damage or changes in the inner membrane organization opening DPA-channels (Setlow, 2003) might be involved in the HPT-mediated non-physiological DPA release (Paidhungat et al., 2002; Wilson et al., 2008). The membrane structure and T,p-dependent phase behavior is influenced by membrane proteins and, vice versa, protein conformation (and function) can be influenced by the lipid environment (Ulmer et al., 2002; Winter and Jeworrek, 2009), which makes it difficult to clearly spot reasons for differences in the release of DPA from spores of different species. Thus, inner membrane properties (e.g., fatty acids, density, rigidity, and phase behavior) as well as the type and abundance of specific membrane proteins could potentially influence the DPA release rate and account for species-specific differences. However, high pressure can counteract fluidizing effects on the inner spore membrane (phase transition from gel state to the liquid-crystalline phase) occurring during heat treatments at ambient pressure (Hofstetter et al., 2013b). Additionally, it was reported that inactivation and DPA release do not require disturbance of the highly ordered membrane state (*C. beijerinckii, C. sporogenes*, 200 or 600 MPa, 90°C, up to 60 min; Hofstetter et al., 2013b). This makes it likely that alterations in the structure of membrane proteins such as channel proteins (Black et al., 2005) or associated DPA binding proteins (SpoVA proteins, Li et al., 2012; Doona et al., 2014) are involved in the inner membrane losing its barrier function in response to HPT treatments at high pressures/elevated temperatures. Thus, differences in such proteins (or their lipid environment) could account for organism-specific differences in non-physiological DPA release rates.
(ii) Although the non-physiological DPA release was reported to be not limited by the activity of the two CLEs conserved among *Bacillus* species (Paredes-Sabja et al., 2011), CwlJ and SleB, such enzymes might play a role in the non-physiological germination pathway. This appears possible since CwlJ (GerQ-dependent) can be activated by DPA release (or exogenous Ca–DPA) and SleB (YpeB-dependent) can be activated by cortex deformation (due to core rehydration). Activation and cortex degradation by such enzymes can facilitate further DPA release and core rehydration (Wuytack et al., 1998; Paidhungat et al., 2002; Setlow, 2003; Black et al., 2007; Reineke et al., 2011, 2013b). *C. botulinum* strain TMW 2.990 (Beluga) possesses SleB (NCBI accession EES50540.1) but lacks YpeB, CwlJ, and GerQ. However, YpeB appears to be required for functional SleB in *Bacillus* and *Clostridium* spores (*C. difficile*, Burns et al., 2010; Cartman and Minton, 2010; Paredes-Sabja et al., 2011). Similar to many *Clostridium* spores, *C. botulinum* TMW 2.990 presumably relies on the exo-acting lytic transglycosylase (Gutelius et al., 2014), SleC (EES49934.1), which is activated by a Csp protease (Adams et al., 2013) for cortex hydrolysis during germination (Paredes-Sabja et al., 2009). Unlike CwlJ and/or SleB, SleC appears not to be activated in response to DPA release or core rehydration (Paredes-Sabja et al., 2008; Wang et al., 2012), which makes it questionable whether it plays a role in non-physiological DPA release from *Clostridium* spores. Thus, similar to *B. subtilis* spores, non-physiological DPA release from *C. botulinum* type E at high pressure/elevated temperatures is likely to be triggered in a CLE-independent manner (Reineke et al., 2013a). However, this would also indicate that the sequential steps of a rapid DPA release, partial core rehydration, CLE activation, cortex lysis, further core rehydration, and inactivation, proposed for *Bacillus* spores treated at high pressure/elevated temperatures, might not occur identically in *C. botulinum* type E spores. Consequently, other molecules/steps might be involved. Provided that SleB is actually not functional and SleC not activated by HPT, there is still the possibility that other, unknown cortex lytic mechanisms (e.g., involving other putative CLEs: EES48735.1 or EES47840.1) are triggered by the release of DPA, directly by HPT, or in consequence of morphological changes in the spore induced by HPT. Finally, it might also be possible that spores are inactivated without the need for cortex degradation.

In contrast to the rapid DPA release at high pressure/elevated temperatures, the effective release of DPA from *C. botulinum* type E spores at high pressure levels (750 MPa) combined with moderate temperatures appears to present no common response in *Bacillus* spores. Again the barrier function of the inner spore membrane and/or the activation of CLEs could potentially play a role in such species-specific differences. However, exact reasons for this striking difference are completely unclear.

Generally, a comparison of DPA release and inactivation data indicates that a rapid and substantial release of DPA after HPT treatments presents a crucial step for an effective spore inactivation, which supports earlier findings for *B. subtilis* spores (Reineke et al., 2013b). However, comparing DPA release and inactivation data between different species (e.g., *B. subtilis* and *C. botulinum* type E spores) also shows that DPA release profiles are helpful to characterize the response of spores to pressure but are not suitable as a measure for HPT resistance properties of spores from different species (Hofstetter et al., 2013a).

### Heat Susceptible Spore Fraction after HPT Treatments

A second heat treatment was employed to estimate the number of spores, which lost their spore-specific heat resistance in response to a HPT treatment but were not inactivated during this treatment. This is likely to apply primarily to physiologic-like germinated spores, which germinated during a HPT treatment and survived it or germinated after a treatment. There were virtually no vegetative cells remaining in the purified spore suspensions prepared for HPT treatments, and it is unlikely that many vegetative cells survive HPT treatments. Therefore, the inactivation of initially present vegetative cells is unlikely to play a role in the observed differences in cell counts between HPT treated and HPT/heat treated samples. However, *C. botulinum* type E spores normally tolerate 60°C but their resistance is much lower than that of other spore formers and closer to that of their vegetative forms. Thus, it cannot be excluded that non-germinated, HPT-damaged but germinable spores are inactivated by a second thermal treatment.

The absence of significant differences between cell counts of HPT and HPT/heat treated samples after any HPT treatment at 60 and 75°C (**Figure 4**) indicates that, regardless of the pressure applied (300–1200 MPa), there are no germinated spores surviving or spores germinating after such HPT teatments. Additionally, this means that such process conditions do not create sublethally damaged but germinable and heat susceptible spores. This also applies to HPT treatments at 750–1200 MPa/30°C and 900–1200 MPa/45°C. This indicates that, despite of the rapid inactivation of a significant number of spores within the population, surviving spores remain largely intact, i.e., retain their heat resistance and viability. This is in accordance with the proposed inactivation mechanism via non-physiological germination, where DPA release, core rehydration and inactivation were proposed to occur in immediate succession at *p* > 600 MPa and T > 60°C (Reineke et al., 2013b).

Small but significant differences, which tended to slightly increase with longer dwell times, were detected after 300–450 MPa/30–45°C treatments. The largest heat susceptible spore fraction [maximum around 0.6 log (N_t_/N_t__+__heat_), **Figure 4**] was detected after 300 MPa/30°C treatments, which matches the accelerated release of DPA under such treatment conditions compared with that after 450 MPa treatments at moderate temperatures (**Figure 3**). This suggests that either pressure-induced physiologic-like germination occurs during such treatments and germination leads to the loss of resistance after the HPT treatment and/or processing conditions are not harsh enough to inactivate spores germinating during a treatment. Very small differences in cell counts of HPT versus HPT/heat treated samples were observed after treatments up to a pressure of 600 MPa at 30°C. The fact that such differences do not tend to increase with prolonged dwell times, might indicate that geminating spores are increasingly physiologically damaged during treatments resulting in their inactivation.

This points toward physiologic-like germination (though not very effective) of *C. botulinum* type E spores triggered by moderate pressure levels of 300–450 MPa (up to 600 MPa) combined with moderated temperatures up to 45°C. This pressure/temperature range overlaps with that previously reported for *B. subtilis*, where the optimum for physiologic-like germination can be found around 80–150 MPa but occurs up to a threshold pressure of 600 MPa (Wuytack et al., 1998; Reineke et al., 2013b). However, the effectiveness of pressure to induce physiologic-like germination of *C. botulinum* type E spores appears to be drastically lower than that of spores from other species [e.g., *B. subtilis* (Reineke et al., 2013b); *G. steorothermophilus* (Georget et al., 2014)]. This low effectiveness and its maximum at low pressures/temperatures is in accordance with an earlier report where 300 MPa treatments at ambient temperature tended to slightly (but not significantly) shorten the time to detect growth from HPT treated *C. botulinum* type E spores (Lenz et al., 2014). Results from other studies suggest that pressure treatments might be generally not very effective in triggering physiologic-like germination of *Clostridium* spores. For example, 100–200 MPa treatments for 7 min did not induce germination of *C. perfringens* spores within 60 min after pressure treatment (Akhtar et al., 2009) and pressure cycling was relatively ineffective in reducing *C. sporogenes* viable spore counts (60 MPa followed by 400 MPa at 60°C, <3 log inactivation, Mills et al., 1998).

### Lysozyme

Small (maximum 0.23 log after 600 MPa/75°C) and in most cases not significant differences between *C. botulinum* type E spore counts in plates with and without lysozyme were exclusively detected at pressure levels above 600 MPa (**Figure 5**). This indicates that HPT treatments below 600 MPa do not provoke the formation of spores with both defects in their cortex lytic machinery and coat layers but without severe damages in other spore components or molecules essential for germination and outgrowth. At 600–750 MPa, a small spore fraction with exactly such injuries can be observed, which tends to be larger when process temperatures are increased and dwell times prolonged. At 900–1200 MPa, the number of spores with such injuries decreases again, which might be due to severe damages in spore components essential for germination and/or outgrowth accompanying injuries in the cortex lytic machinery and coats. However, in the latter pressure range, inactivation levels are high, i.e., nearly complete inactivation, and difficulties in detecting differences in cell counts might play a role.

The occurrence of lysozyme-dependently germinable spore fractions after intense HPT treatments is generally in accordance with the previously described prolongation of the times to detect growth from high pressure/high temperature treated *C. botulinum* type E spores (Lenz et al., 2014), which is accompanied by an increase in the heterogeneity of detection times among individual spores and indicative of sublethal damages in the germination machinery. The result that inactivation levels (**Figure 3**) do not correlate with lysozyme susceptible spore fractions (**Figure 4**) corroborates earlier findings for *B. subtilis*, i.e., that the inactivation of CLEs does not present a prerequisite for spore inactivation (Reineke et al., 2011).

In contrast to the very weak effect of lysozyme on the recovery of HPT treated spores observed here, lysozyme was reported to significantly aid in the recovery of heat treated *C. botulinum* type E spores (Alderton et al., 1974; Lindstrom et al., 2003). For example, 10 μg/mL lysozyme in the plating medium resulted in the detection of 4 log spores/mL instead of a complete inactivation of initially 6–8 log spores/mL determined on plates without lysozyme after heat treatments at 85°C for 10 min [*C. botulinum* TWM 2.990 (Beluga), Peck et al., 1992]. This suggests that damages in the cortex lytic machinery and the coat provoked by HPT and heat alone are different and/or that such damages are more likely to occur concomitant with inactivation (due to severe injury of other spore components) during HPT compared with heat treatments. This is in accordance with the significantly different inactivation mechanisms proposed and spore resistance factors important for heat and HPT inactivation (Setlow and Johnson, 2013).

Notably, it was reported later that lysozyme addition to liquid recovery medium for MPN counts improves only the initial (4 days) recovery of heat and high pressure injured *C. botulinum* type A and B spores and has no significant effect when added to plating agar (Reddy et al., 2010). This appears to point toward distinct *C. botulinum* type- and strain-dependent and/or treatment intensity-related differences. Another explanation for the comparably low effect of lysozyme addition found here for HPT treated and earlier for heat treated spores (Alderton et al., 1974; Peck et al., 1992; Lindstrom et al., 2003) might be found in the recovery duration, which has not been specified in some earlier studies.

### Isoeffect Curves

Isoeffect curves for the inactivation of *C. botulinum* TMW 2.990 spores by 1 and 3 log cycles (**Figure 7**) demonstrate that either high pressures levels at low temperatures (e.g., 1 log inactivation after 600 MPa/30°C/10 min) or lower pressure levels at higher temperatures (e.g., 1 log inactivation after 400 MPa/80°C/10min) can be used to achieve an inactivation of 90 and 99.9%, respectively (initially ∼10^7^ viable spores/mL). Such inactivation levels are far below the 6 log inactivation stipulated for the inactivation of non-proteolytic *C. botulinum* spores for the production of safe food (Peck et al., 1995; Garcia-Graells et al., 2002). However, the shape of isoeffect curves as graphical representation of the estimated parameters can facilitate the description of the pressure- and temperature-dependent behavior of *C. botulinum* type E during HPT processing.

The pressure/temperature-dependency of spore inactivation during a given dwell time appeared to be almost linear and was not drastically altered when HPT treated spores were subjected to a second heat treatment or when lysozyme was added to the recovery medium. This reflects the fact that no large heat susceptible or lysozyme-dependently germinable spore fractions were detected after HPT treatments. Accordingly, the p/T/t parameter range displayed in **Figure 7** is not suitable for efficiently triggering pressure-induced physiologic-like germination or provoking damages in the cortex lytic machinery and coat layers leaving other spore components essential for germination and outgrowth intact (both discussed above).

Similar to inactivation, the p/T dependency of a 90% DPA release appears to be almost linear, at least for longer dwell times. This means that either high pressures levels at low temperatures or low pressure levels at high temperatures applied for a specific dwell time trigger the release of the same amount of DPA (**Figure 8**). Isoeffect lines for 1 log, i.e., 90%, inactivation and those for a 90% DPA release after 5–10 min would intersect at points corresponding to a process temperature of around 60–70°C at around 500 MPa. Above this temperature range, DPA release occurs significantly faster than inactivation. At temperatures below 60°C, inactivation occurs faster than the release of DPA, i.e., DPA release appears to be less important for inactivation. Notably, this matches the process conditions where inactivation of *C. botulinum* type E is significantly accelerated. This indicates that a rapid release of DPA is a crucial step for the effective inactivation of *C. botulinum* type E. However, a direct comparison of concrete calculated data points of isoeffect lines for a 1 log inactivation and those for a 90% DPA release is impeded by two facts. Firstly, the inactivation data refer to a 90% loss of viability of the initially viable spore fraction, whereas DPA release data refer to a 90% release of the total amount of available DPA, i.e., from the core of all spores present including viable and dormant spores. Secondly, inactivation data used for modeling excludes, whereas DPA release data includes effects occurring under non-isobaric and non-isothermal conditions, i.e., pressure build-up and release. Nonetheless, the different slopes of isoeffect curves for inactivation and DPA release indicate that an increase in the process temperature accelerates DPA release markedly more than inactivation. This is indicative of a rapid DPA release and subsequent inactivation at high process temperatures versus a slower release of DPA in relation to the inactivation rate at lower process temperatures. This putatively reflects the presence of different, p/T-dependent mechanisms underlying the inactivation of *C. botulinum* type E spores.

In comparison with the well-characterized spore forming model organism *B. subtilis* (Reineke et al., 2012) markedly different shapes of isoeffect inactivation curves can be observed, which reflect significant differences in the resistance of the two organisms against HPT treatments. Whereas *C. botulinum* type E requires higher pressure levels at high temperatures to be inactivated, *B. subtilis* but not *C. botulinum* type E inactivation is retarded at high pressure/moderate temperatures (below ∼60°C). Similarly, isoeffect curves for DPA release are different between these organisms (Reineke et al., 2013b). In contrast to *B. subtilis*, low pressure levels appear to be less effective in triggering the release of DPA from *C. botulinum* type E spores and the role of the pressure level in triggering DPA release is not diminished above a certain threshold pressure (600 MPa for *B. subtilis*). Additionally, no distinct p/T zones with markedly different shapes of DPA release isoeffect curves were detected for *C. botulinum* type E. However, the latter could be related to the fact that the effect of pressure levels below 300 MPa and prolonged dwell times exceeding 10 min was not investigated here.

However, data obtained here together with the inactivation mechanism proposed for the model organism *B. subtilis* (Reineke et al., 2013a) suggest that p/T combinations provoking physiologic-like and non-physiological germination are very similar for *C. botulinum* type E and *B. subtilis*. Although HPT is markedly less effective in triggering physiologic-like germination, HPT treatments at 300–450 MPa/30–45°C (up to 600 MPa/30–45°C, possibly up to 750 MPa/45°C) are likely to induced physiologic-like germination with the effectiveness tending to increase with decreasing pressure and temperature as well as prolonging dwell times. The generally low effectiveness of pressure to induce physiologic-like germination after treatments for up to 10 min dwell time at mild processing conditions can be assumed to account for the very low inactivation of *C. botulinum* type E spores after treatments in this parameter range. At >500 MPa/>60–70°C, non-physiological germination, i.e., rapid DPA release putatively due to the inner membrane losing their barrier function, might be responsible for the effective inactivation of *C. botulinum* type E spores observed at such high pressure/elevated temperatures. In contrast to *B. subtilis*, a considerable portion of a *C. botulinum* type E spore population can be inactivated at pressure levels over 500 MPa and temperatures below 60°C. At such parameter combinations, DPA release appears to play a less important role for inactivation compared with high pressure/high temperature treatments. This suggests that damages to the inner membrane barrier function are less severe and injury of additional spore components essential for germination and outgrowth might be involved.

### Strain Comparison

The general order of the HPT resistance of spores from *C. botulinum* type E strains TMW 2.990 > TMW 2.992 > TMW 2.994 (**Figure 9**) is in accordance with that found in an earlier study (Lenz and Vogel, 2014). Only slight variations in the maxima of strain-dependent differences can be found between this (∼2 log cycles) and an earlier study (∼2.5 log cycles, 800 MPa/80°C/600 s, Lenz and Vogel, 2014) where the same strains were used. Thus, the general order of the HPT resistance of different *C. botulinum* type E strains can be observed independently from differences in the process design (isothermal–isobaric dwell times considered here), sporulation conditions [SFE medium (cf. Lenz and Vogel, 2014) and sporulation at suboptimal growth temperature (cf. Lenz and Vogel, 2015)], and maximum process intensity (750 MPa/75°C here vs. 800 MPa/80°C; Lenz and Vogel, 2014). A similar range of strain-dependent differences (around 2 log cycles) was reported earlier for *C. botulinum* type E strains Beluga (TMW 2.990) and Alaska (827 MPa/40 and 45°C/5 min, Reddy et al., 1999).

Although comparable data is scarce and the HPT resistance of only a small number of *C. botulinum* type E strains has been investigated up to now, strain-dependent differences observed so far are significantly smaller compared with those reported for other *C. botulinum* serotypes. For example, inactivation of 5.5 log (detection limit) of proteolytic *C. botulinum* type A spores by HPT treatments at 600 MPa/80°C was not reached within 60 min for a resistant strain (TMW 2.365) but within 12 min for a sensitive strain (ATCC 25765; in mashed carrots, Margosch, 2004; Margosch et al., 2004a). Furthermore, log inactivation levels of spores of non-proteolytic *C. botulinum* type B strains, which appear to be generally somewhat less resistant than spores of proteolytic type A, were reported to vary between 0.4 (resistant strain KAP9-B) and >5.5 log cycles (sensitive strain KAP8-B) after 827 MPa/75°C treatments (phosphate buffer, pH 7, Reddy et al., 2006).

The observable trend that strain-dependent differences in HPT inactivation levels differ depending on the p/T range applied (see Strain-Specific HPT Resistance and DPA Content) might just reflect the fact that strain-specific differences become more easily detectable when the process intensity and therefore inactivation levels are increased. However, since maximum differences were observed in a p/T range where rapid non-physiological DPA-release might become the governing factor (>500 MPa/60°C; see Isoeffect Curves), an increase in such strain-dependent differences might be related to strain-specific differences in compartments modulating the non-physiological release of DPA. On the other hand, small differences at low pressure levels combined with low temperatures might indicate that the relatively low susceptibility to undergo pressure-induced physiologic-like germination (see Isoeffect Curves) is conserved among the three strains tested. This might point toward a high similarity in spore compartments that are thought to influence the effectiveness of pressure triggering physiologic-like germination such as the spores’ nGRs.

Dipicolinic acid levels of *C. botulinum* strains TMW 2.990, 2.992, and 2.994 were 155 ± 23, 132 ± 9, and 72 ± 14 amol DPA/spore, respectively. These values are substantially lower than those previously reported for *B. subtilis* spores (365, Hindle and Hall, 1999; 174–384, Huang et al., 2007; 193, Fichtel et al., 2007a; 216 amol/spore, Sanchez-Salas et al., 2011), various *Bacillus* spores (193–505, Fichtel et al., 2007a; 365–820 amol/spore, Kort et al., 2005), and *Clostridium* spores (598 *C. hungatei*, 614 *C. sporogenes*, 747 *Clostridium* G5A-1, Yang et al., 2009). Earlier studies reporting on the DPA content of *C. botulinum* spores specified a range between 8.7–13.4% DPA/spore dry weight for type A, 7.4–8.1% for type B, and 9.5% for type E (Grecz and Tang, 1970). This is completely in the range assumed to be present in *B. subtilis* spores but contrasts the range of 3–4% DPA reported somewhat earlier for *C. botulinum* type A (Day and Costilow, 1964). Differences in the determined DPA level might occur due to various influence factors including the accuracy of the method used, species- and strain-specific differences, and sporulation conditions. However, a direct comparison with the spores’ DPA content found here is not possible, since the spore’s dry mass was not determined. Generally, a comparison between the DPA content and the HPT resistance of the different strains used here corroborates results from various previous studies where it was shown that the DPA content of spores does not necessarily correlate with their HPT resistance (e.g., Reineke et al., 2011).

## Conclusion

Commonalties and significant differences exist between the HPT-mediated inactivation of *C. botulinum* type E and other endospores. In accordance with findings for other spore formers, data obtained here suggests that:

– the ability to retain DPA rather than its amount present in the spore core is important for HPT resistance,
– the activity of CLEs is likely to be not required for a rapid, non-physiological DPA release from *C. botulinum* type E, and
– p/T zones with differences in the inactivation mechanism appear to largely overlap between *B. subtilis* and *C. botulinum* type E, where a physiologic-like germination at 300 – 450 (600) MPa/30–45°C and a non-physiological germination at >500 MPa/>60–70°C are likely to occur.

In contrast to spores from other species, data for *C. botulinum* type E suggests that

– the efficiency of low pressure/moderate temperature treatments to induce physiologic-like germination appears to be markedly lower than that reported for *B. subtilis* (but similar to other *Clostridium* spores),
– inactivation and DPA release at >500 MPa/>60–70°C occurs significantly faster,
– inactivation at >500 MPa/<60–70°C is not completely retarded (but also not very effective), and
– the extent of strain-dependent differences in inactivation levels seems to be generally less pronounce, which especially applies to HPT conditions inducing germination via the activation of nGRs (moderate pressure/moderate temperature), but becomes larger under conditions where non-physiological germination can be assumed to occur (high pressure/elevated temperatures).

Although synergistic or antagonistic effects on the HPT inactivation of *C. botulinum* type E spores occurring in complex food matrices cannot be estimated based upon the data presented here, it appears that HPT can be used to efficiently inactivate *C. botulinum* type E spores. No problematic tailing effects (almost first order kinetics) or pressure zones stabilizing spores were identified (at least within 10 min dwell time). However, the very low effectiveness of pressure-induced physiologic-like germination of *C. botulinum* type E (and other *Clostridium*) spores at low pressures and temperatures makes two-step approaches unsuitable for an industrial application.

Finally, much more work should to be conducted to better understand the inactivation mechanism of bacterial endospores in general and *C. botulinum* spores in particular, e.g., to identify the exact molecular mechanisms of HPT-induced DPA release, to clarify the role of CLEs in HPT-induced non-physiological germination, and to further investigate the single spore fate in heterogeneous populations.

## Conflict of Interest Statement

The authors declare that the research was conducted in the absence of any commercial or financial relationships that could be construed as a potential conflict of interest.
