# Supplementary material for: High pressure thermal inactivation of Clostridium botulinum type E endospores – kinetic modeling and mechanistic insights
Source: Front Microbiol. 2015 Jul 3;6:652. doi: 10.3389/fmicb.2015.00652 (PMC4490342; doi:10.3389/fmicb.2015.00652)
Supplement: Supplementary file 1 [file Table_1.DOCX]

Supplement Table 1: Summary of parameters for the Weibull model and the Taylor series as well as the confidence intervals.

| Inactivation | | | | | Heat treatment | | | | Plated on lysozyme | | | |
| --- | --- | --- | --- | --- | --- | --- | --- | --- | --- | --- | --- | --- |
| Parameter | Value | Std | 95% Confidence | | Value | Std | 95% Confidence | | Value | Std | 95% Confidence | |
| a | 0.02397187 | 0.04112893 | -0.06906822 | 0.11701197 | 0.03220063 | 0.04170704 | -0.06214725 | 0.12654852 | 0.03178821 | 0.03954437 | -0.05766737 | 0.1212438 |
| b | -0.00010637 | 0.00013122 | -0.00040322 | 0.00019047 | -0.00010579 | 0.00013307 | -0.00040681 | 0.00019523 | -9.54E-05 | 0.00012617 | -0.00038077 | 0.00019005 |
| c | 0.00019127 | 0.00156994 | -0.00336017 | 0.00374272 | -0.00021685 | 0.001592 | -0.0038182 | 0.0033845 | -0.00027502 | 0.00150945 | -0.00368963 | 0.00313959 |
| d | 1.23E-07 | 1.65E-07 | -2.50E-07 | 4.97E-07 | 9.31E-08 | 1.67E-07 | -2.86E-07 | 4.72E-07 | 6.80E-08 | 1.59E-07 | -2.91E-07 | 4.27E-07 |
| e | -5.04E-06 | 1.62E-05 | -4.18E-05 | 3.17E-05 | -8.61E-07 | 1.65E-05 | -3.81E-05 | 3.64E-05 | 9.24E-07 | 1.56E-05 | -3.44E-05 | 3.62E-05 |
| f | -7.81E-07 | 3.07E-06 | -7.73E-06 | 6.16E-06 | -2.10E-07 | 3.11E-06 | -7.25E-06 | 6.83E-06 | -2.57E-07 | 2.95E-06 | -6.93E-06 | 6.42E-06 |
| g | 3.89E-10 | 2.57E-09 | -5.42E-09 | 6.20E-09 | 8.42E-10 | 2.61E-09 | -5.05E-09 | 6.74E-09 | 1.39E-09 | 2.47E-09 | -4.20E-09 | 6.98E-09 |
| h | 1.54E-08 | 3.37E-08 | -6.08E-08 | 9.16E-08 | 6.78E-09 | 3.42E-08 | -7.05E-08 | 8.41E-08 | 1.77E-09 | 3.24E-08 | -7.15E-08 | 7.51E-08 |
| Adj R^2^ | 0.95 | | | | 0.94 | | | | 0.95 | | | |
